# Human urine-derived stem cells protect against renal ischemia/reperfusion injury in a rat model via exosomal *miR-146a-5p* which targets *IRAK1*

**DOI:** 10.7150/thno.42153

**Published:** 2020-07-25

**Authors:** Xirui Li, Jun Liao, Xiaojun Su, Weiqiang Li, Zirong Bi, Jiali Wang, Qun Su, Huiting Huang, Yongcheng Wei, Yifang Gao, Jun Li, Longshan Liu, Changxi Wang

**Affiliations:** 1 Organ Transplant Center, The First Affiliated Hospital of Sun Yat-sen University, Guangzhou, Guangdong, People's Republic of China.; 2 Center for Stem Cell Biology and Tissue Engineering, Key Laboratory for Stem Cells and Tissue Engineering, Ministry of Education, Sun Yat-Sen University, Guangzhou, Guangdong, People's Republic of China.; 3 Department of Nephrology, The First Affiliated Hospital of Sun Yat-sen University, Guangzhou, Guangdong, People's Republic of China.; 4 Guangdong Provincial International Cooperation Base of Science and Technology (Organ Transplantation), The First Affiliated Hospital of Sun Yat-sen University, Guangzhou, Guangdong, People's Republic of China.; 5 Guangdong Provincial Key Laboratory on Organ Donation and Transplant Immunology, Guangzhou, Guangdong, People's Republic of China.

**Keywords:** urine-derived stem cells, ischemia/reperfusion injury, exosomes, *miR-146a-5p*, * IRAK1*

## Abstract

**Rationale:** Ischemia/reperfusion injury (IRI) is a major cause of acute kidney injury (AKI) that is associated with high morbidity and mortality, and for which specific treatments are lacking. In this study, we investigated the protective effect of human urine-derived stem cells (USCs) and their exosomes against IRI-induced AKI to explore the potential of these cells as a new therapeutic strategy.

**Methods:** USCs were derived from fresh human urine. Cell surface marker expression was analyzed by flow cytometry to determine the characteristics of the stem cells. Adult male Sprague-Dawley rats were used to generate a lethal renal IRI model. One dose of USCs (2×10^6^ cells/ml) or exosomes (20 µg/1 ml) in the experimental groups or saline (1 ml) in the control group was administered intravenously immediately after blood reperfusion. Blood was drawn every other day for measurement of serum creatinine (sCr) and blood urea nitrogen (BUN) levels. The kidneys were harvested for RNA and protein extraction to examine the levels of apoptosis and tubule injury. *In vitro*, the hypoxia-reoxygenation (H/R) model in human kidney cortex/proximal tubule cells (HK2) was used to analyze the protective ability of USC-derived exosomes (USC-Exo). Quantitative reverse-transcriptase polymerase chain reaction (qRT-PCR), western blotting, superoxide dismutase activity, and malonaldehyde content analyses were used to evaluate oxidative stress in HK2 cells treated with USC-Exo after H/R. Exosomal microRNA sequencing techniques and bioinformatics analysis were used to search for enriched miRNAs in the exosomes and interacting genes. The interaction between miRNAs and the 3' untranslated region of the target gene was detected using a dual luciferase reporting system. The miRNA mimic and inhibitor were used to regulate the miRNA level in HK2 cells.

**Results:** Treatment with USCs led to reductions in the levels of sCr, BUN, and renal tubular cell apoptosis; inhibited the infiltration of inflammatory cells; and protected renal function in the rat IRI model. Additionally, USC-derived exosomes protected against IRI-induced renal damage. *miR-146a-5p* was the most abundant miRNA in exosomes obtained from the conditioned medium (CM) of USCs. *miR-146a-5p* targeted and degraded the 3'UTR of* interleukin-1 receptor-associated kinase 1 (IRAK1)* mRNA, subsequently inhibited the activation of nuclear factor (NF)-κB signaling, and protected HK2 cells from H/R injury. USC transplantation also upregulated *miR-146a-5p* expression, downregulated *IRAK1* expression and inhibited nuclear translocation of NF-κB p65 in the kidney of the rat IRI model.

**Conclusions:** According to our experimental results, USCs could protect against renal IRI via exosomal *miR-146a-5p*, which could target the 3'UTR of *IRAK1* and subsequently inhibit the activation of NF-κB signaling and infiltration of inflammatory cells to protect renal function. As a novel cell source, USCs represent a promising non-invasive approach for the treatment of IRI.

## Introduction

Acute kidney injury (AKI) often leads to rapid renal dysfunction and is associated with high mortality and morbility 1. Renal ischemia/reperfusion injury (IRI) is a primary cause of AKI 2. Deprivation and subsequent restoration of blood flow and oxygenation induce IRI, which mainly occurs after infarction, sepsis, and organ transplantation 3. Many physiopathological events are involved in IRI, including reactive oxygen species (ROS) generation, neutrophil infiltration, and inflammatory cytokine production 4, 5. There is a lack of effective treatments for IRI-induced AKI in clinic, and thus, an urgent unmet need for innovative and effective therapies 6, 7.

Stem cell therapy represents a promising new frontier in the treatment of renal diseases 8. Many types of stem cells, including mesenchymal stem cells (MSCs), spermatogonial stem cells, induced pluripotent stem cells (iPSCs) and the cells derived from iPSCs have been found to either attenuate kidney dysfunction or stabilize morphological changes in renal IRI 9, 10. Treatments with MSCs specifically have entered clinical trials 11. Recently, human urine-derived stem cells (hUSCs) were introduced as a new promising candidate in many applications, such as vascular and neural tissue engineering, cartilage regeneration, and bladder/ureter remodeling 12-16. USCs, which are isolated from voided urine, exhibit many characteristics of MSCs and can differentiate along multiple cell lineages to generate endothelial cells, neurocytes, chondrocytes, and myocytes 17. Also, some cell characteristics of USCs will change under the disease state. The patient-derived USCs could be used as a tool to predict the outcome of the kidney disease 18. Additionally, USCs possess the advantages of being easily accessible, consistently producible, and relatively free of ethical concerns in comparison to stem cells from other donor sources 19. Therefore, USCs are emerging as an attractive cell source for a variety of therapies 20.

The therapeutic efficacy of USCs against IRI has been reported; however, the underlying mechanism remains incompletely understood 21. The mechanisms responsible for the beneficial effects of stem cell therapies include both direct pathways (e.g., stem cells may be directly incorporated into injury tissue) and indirect pathways (e.g., stem cells may secrete paracrine factors particularly through production of extracellular vesicles [EVs]) 22. Among the various types of EVs, exosomes are 30- to 150 nm cell membrane-containing vesicles that are secreted by almost all cell types. One study reported that MSC-derived exosomes can mediate repair of kidney injury 23, 24. USCs also secrete exosomes, which were shown to protect against diabetic nephropathy 25, promote of angiogenesis and wound healing in diabetic wound repair 26, and promote the angiogenesis and muscle regeneration in hind-limb ischemia 27. It is well known microRNAs (miRNAs) or small noncoding RNAs in exosomes play important roles in the regulation of target genes 28. We herein hypothesized miRNAs in USC-derived exosomes (USC-Exo) might be vital for the USC-mediated protection against IRI-induced renal injury.

In the present study, we investigated the potential renal protective effect of USCs and their exosomes using *in vivo* and *in vitro* models of IRI. We also explored the underlying mechanism of this protective effect by analyzing the miRNAs present in USC-Exo to gain new insights for the application of USCs in relevant therapies.

## Materials and Methods

### Preparation of USCs

USCs were isolated from two healthy adults. This study was approved by the institutional review board of medical ethics, and written informed consent was obtained from urine donors. A total of 100 ml fresh urine from each healthy adult was collected in one experiment. The urine sample was centrifuged at 400×*g* for 10 min, and the obtained pellet was washed twice with phosphate-buffered saline (PBS). The cells were resuspended in fresh USC medium and seeded in 48-well plates. The cells were allowed to grow for 4 days, and the culture medium was replaced gently. By day 7, the cells had formed tiny colonies. After culture for another week, USCs were passaged and expanded. USCs of passages 3-5 (P3-P5) were collected for use in the experiments in this study. USC medium consisted of medium A and medium B at a 1:1 ratio. Medium A consisted of high-glucose Dulbecco's Modified Eagle's Medium (DMEM; HyClone) + non-essential amino acid (NEAA) solution (Gibco) + GlutaMAX (Gibco) + 10% fetal bovine serum (FBS; Gibco). Medium B consisted of SingleQuot Kit CC-3191 & CC-4172 renal epithelial cell growth medium (REGM) supplements (Lonza). The expression levels of cell surface markers and specific genes were analyzed by flow cytometric analysis to confirm the characteristics of USCs.

### Animal model of IRI

A lethal renal IRI model was established in male adult Sprague-Dawley rats (200-250 g) purchased from Beijing Vital River Laboratory Animal Technology Co., Ltd. The rats were anesthetized with intraperitoneal pentobarbital (4 mg/kg). The right kidney was removed, and the left renal pedicle was then clamped for 45 min to induce ischemia. Normal body temperature was maintained using a rectal temperature probe and heat supply. Upon reperfusion of the left kidney, USCs (2×10^6^ cells in 1 ml saline), exosomes (20 μg/ml saline), or saline (1 ml) were injected into the rat via the dorsal vein of the penis. Animals were housed in pathogen-free conditions under a controlled 12-h light-dark cycle and allowed free access to water and chow. Survival rates, serum creatinine (sCr) and blood urea nitrogen (BUN) levels, and renal histopathology were assessed at the designated time points. Renal damage was scored according to the following criteria: 1 = damage area < 25%; 2 = damage area 25-50%; 3 = damage area 50-75%; 4 = damage area >75% 29.

All experiments were conducted in strict accordance with the institutional policies of the First Affiliated Hospital of Sun Yat-sen University. All efforts were made to minimize animal suffering. Animals were housed in pathogen-free conditions with a controlled 12 h light-dark cycle environment and allowed free access to water and chow. This study was approved by the Animal Ethics Committee of the First Affiliated Hospital of Sun Yat-sen University.

### *In vitro* model of hypoxia/reoxygenation (H/R)

HK2 cells (human kidney cortex/proximal tubule cells) were purchased from American Type Culture Collection (ATCC® CRL-2190™). HK2 cells were cultured in DMEM/Nutrient Mixture F12 (DMEM/F12, Gibco) supplemented with 10% FBS, 500 U/ml penicillin, and 500 μg/ml streptomycin. For all culture conditions, the cells were incubated in a humidified incubator containing 5% CO_2_ at 37 °C. The H/R process was optimized according to the previously described methods 30. H/R injury was induced in normal HK2 human kidney cortex/proximal tubule cells by incubation in glucose- and serum-free medium in a 1% O_2_ environment for 48 h followed by reoxygenation with normal O_2_ during culture with USC conditioned medium or medium containing exosomes for 6 h.

### Terminal deoxynucleotidyl transferase dUTP nick end labeling (TUNEL) assay

Renal sections were stained with the *In situ* Cell Death Detection Kit (11684817910, Roche) according to the manufacturer's protocol. Briefly, each slide was deparaffinized, rehydrated, and treated with proteinase K (20 mg/L) for 10 min at 37 °C. Endogenous peroxidase was inhibited by treatment with 3% hydrogen peroxide for 5 min, and then the slides were incubated in the TUNEL reaction mixture containing terminal deoxynucleotidyl transferase (TdT) and digoxigenin-11-dUTP for 2 h at 37 °C. Next, the 3,3-diaminobenzidine chromogen was applied, and finally, the number of apoptotic cells was analyzed quantitatively by counting of TUNEL-positive cells in three randomly selected fields of view under a microscope.

### Measurement of malondialdehyde (MDA) content and superoxide dismutase (SOD) activity

MDA content and SOD activity were measured in renal tissues and HK2 cells to assess the level of oxidative stress. The intracellular concentration of MDA was detected using a MDA assay kit (TBA Method, Nanjing Jiancheng Bioengineering Institute) and was reported as nmol/mg of extracted protein. SOD activity was detected using the Total Superoxide Dismutase (T-SOD) assay kit (Hydroxylamine method, Nanjing Jiancheng Bioengineering Institute) and was reported as U/mg protein.

### Western blot analysis

For western blotting, the soluble proteins were separated on 12% Tris-glycine gels and transferred onto polyvinylidene difluoride (PVDF) membranes (Millipore). Protein gel electrophoresis and protein transfer were performed according to standard procedures. Non-specific binding was blocked by addition of 5% non-fat milk for 1 h at room temperature (RT). Then membranes were incubated with specific primary antibodies in a humidified box at 4 °C overnight. After washing with 0.1% Tween-20 in PBS three times, the membranes were incubated with the appropriate horseradish peroxidase (HRP)-conjugated secondary antibody for 1 h at RT. Protein bands were detected using the enhanced chemiluminescence technique (GE AI600, USA). The antibodies employed in the experiment are listed in Table 1.

### Quantitative reverse transcription-polymerase chain reaction (qRT-PCR) analysis

qRT-PCR was performed using the 2*Super SYBR Green qPCR Master Mix (ES Science) according to the manufacturer's instructions. The reaction volumes contained 8 μl diluted cDNA solution, 10 μl Mix, and 2 µl each of forward and reverse primer. qRT-PCR was performed on the CFX96 touch (BIO-RAD) with the following cycling scheme: 10 min at 95 °C followed by 40 cycles of 15 sec at 95 °C and 60 sec at 60 °C. Ct-values were calculated with automatically set thresholds and baselines, and those higher than 38 were excluded from the analysis. The primers used for qRT-PCR are listed in Table 2.

### Exosome isolation from CM or serum

During cell passage, digestion was stopped with exosome-depleted FBS (System Biosciences, SBI). Briefly, when the cells reached 80% confluency, they were digested with 0.25% EDTA-trypsin, centrifuged at 200×*g* for 5 min, and washed with medium supplemented with exosome-free FBS (SBI). After washing, 5×10^5^ cells were seeded in 10 cm plates. After 3 days in culture, the supernatant of the culture medium, USC conditioned medium (CM), was collected for exosome separation by the co-precipitation method (ExoQuick-TC, SBI). Before isolation, the culture medium was centrifuged at 3,000×*g* for 15 min to remove dead cells and cell debris. Next, the supernatant was collected, mixed well with a 1/5 volume of the exosome isolation solution, and kept at 4 °C overnight. Next, the mixture was centrifuged at 1,500×*g* at 4 °C for 30 min, and the supernatant was discarded. The pellet of exosomes was resuspended in saline and stored at -80 °C. The protein concentration of exosomes was determined by the BCA protein assay kit (UBIO, UW0202A). The morphology of exosomes was examined by transmission electron microscopy (TEM), and the mean diameter of the exosomes was measured by particle size analysis.

Serum exosomes were extracted with according to the instructions of the serum exosome extraction kit (EXOQ5A-1, SBI). Briefly, 500 μl serum was centrifuged at 3,000×*g* for 15 min to remove cells and cell debris. The supernatant was collected, mixed well with 126 μl exosome precipitation solution, and kept at 4 °C for 30 min. Next, the mixture was centrifuged at 1,500×*g* at 4 °C for 30 min, and the supernatant was discarded. The exosome precipitate was lysed using a specific exosome RNA purification kit (EZBioscience, EZB-exo-RN1), and RNA extraction was carried out according to the kit instructions. Because the amount of RNA obtained from the serum exosomes was very small, we reverse transcribed all the extracted RNA and diluted it 2 times for qRT-PCR analysis.

### *In vitro* exosome tracing

The PKH67 Green Fluorescent Cell Linker Mini Kit (Sigma-Aldrich, Mini67-1KT) was used to stain the membrane of isolated exosomes, and DAPI (4',6-diamidino-2-phenylindole) was used to stain HK2 cell nuclei. All dyeing steps were carried out according to the protocols recommended by the manufacturers. The stained exosomes in Diluent C were extracted again using the exosome extraction kit (as mentioned above). Fluorescently labeled exosomes were added to the supernatant of HK2 cells. After incubation for 30 min, 1 h, or 3 h, the supernatant was discarded and the cells were washed three times with PBS. The fluorescence signals among the adherent cells were detected by fluorescence microscopy to determine whether fusion had occurred between the added exosomes and plated cells.

### miRNA isolation and high-throughput sequencing

To perform miRNA-sequencing analysis, exosomal total RNA was extracted using the miRNeasy Mini Kit (Qiagen). The quality and purity of the extracted RNA were determined using the Agilent 2100 Bioanalyzer (Agilent Technologies). The miRNA sequencing library was obtained using the NEXTflex®Small RNA-Seq Kit v3 (Bioo Scientific Corp, NOVA-5132-05), and we used a sample input of 20 ng total RNA. Finally, we profiled the expression of miRNAs in the library using Hiseq2500 (Illumina, Inc.).

### Cell transfection and dual-luciferase reporting system

HEK293T cells were plated at 1×10^5^ cells/well in a 24-well plate, and transfection was performed once the cells achieved 60-70% confluency. Thirty minutes before transfection, the complete medium was replaced with serum-free medium. Lipofectamine 2000 (Invitrogen Life Technologies, 780373) was used to transfect the cells. The cells were lysed 48 h after transfection, and miRNA expression was detected by chemiluminescence using the Dual-Luciferase Reporter Assay System (Promega). With firefly luciferase as the internal reference, the fluorescence ratio was calculated by dividing the relative light units (RLU) for Renilla luciferase by the RLU for firefly luciferase. The inhibitory effect of the miRNA on target gene expression was compared according to the obtained ratio.

### Fluorescence *in situ* hybridization (FISH)

For FISH analysis, tissue samples were harvested, washed, and immediately placed in the fixative solution for 12 h. After gradient alcohol dehydration and paraffin embedding, tissue slices were cut and then baked in a 62 °C oven for 2 h. Subsequently, the tissue sections were immersed in dimethylbenzene xylene I for 15 min, dimethylbenzene xylene II for 15 min, anhydrous ethanol I for 5 min, anhydrous ethanol II for 5 min, dry naturally and soak in diethylpyrocarbonate (DEPC)-treated water. Then the slices were boiled in the repair solution for 15 min and cooled naturally. Protease K (20 µg/ml) was added for protein digestion at 37 °C for 30 min. After washing with pure water, the slices were washed three times with PBS for 5 min. Pre-hybridization solution was added and incubated at 37 °C for 1 h. After removal of the pre-hybridization solution, the hybridization solution containing an 8 ng/µl *miR-146a-5p* probe was dropped onto the slides for hybridization at 37 °C overnight. Washing with 2*SSC in 37 °C for 10 min, 1*SSC in 37 °C for 10 min, 0.5*SSC in 37 °C for 10 min after hybridization. After the solution was removed, the sealing solution (bovine serum albumin, BSA) was added for incubation at RT for 30 min. After removal of the sealing solution, anti-DIG-488 solution was added for incubation at 37 °C for 50 min. Next the slices were washed with PBS four times, 5 min each, and DAPI solution was added for incubation in darkness for 8 min. After final rinsing, anti-fluorescence quenching sealant was added to seal the slices. The slices were observed under a fluorescence microscope (Olympus), and images were collected.

### Immunofluorescence staining

For immunofluorescence analysis, HK2 cells were seeded on coverslips in 24-well plates. After H/R injury, the cells were washed with PBS twice and fixed in 4% paraformaldehyde at RT for 30 min. After washing with PBS for 5 min to quench the aldehyde group, the cells were permeabilized with PBS containing 0.2% Triton X-100 for 10 min at RT. After washing twice with PBS, the cells were blocked with 10% normal goat serum in PBS for 60 min at RT with gentle shaking. The cells were then incubated with primary NF-κB p65 antibody (CST, 8242S, 1:50 dilution) at 4 °C overnight with gentle shaking. The next day the cells were washed with PBS containing 0.05% Tween 20 and 1% bovine serum albumin (BSA) three times with shaking before incubation with secondary antibody (Alexa Fluor 488-conjugated goat anti-rabbit IgG, ABclonal, AS053, 1:100 dilution) at RT for 1 h in darkness with gentle shaking. The cells were again washed with PBS containing 0.05%Tween 20 and 1% BSA three times with shaking and then incubated in 0.2 mg/ml DAPI solution for 2 min for nuclear staining, followed by a final rinse with PBS. Immunofluorescence staining of NF-κB p65 within the cells was observed under a fluorescence microscope (Olympus).

### Statistical analysis

Data are presented as mean ± standard error of the mean (SEM). First, we determined whether the data followed a normal distribution. When data for two independent conditions followed a normal distribution, the parameter test was used. When variance was homogeneous, the unpaired Student's t test was used. For the comparison of data among multiple groups, one-way analysis of variance (ANOVA) was applied. All of the statistical tests were performed using GraphPad 7.0 (GraphPad Software), and the level of significance was set at *P* < 0.05 for all comparisons.

## Results

### hUSCs were isolated from human urine samples

**Figure 1:**
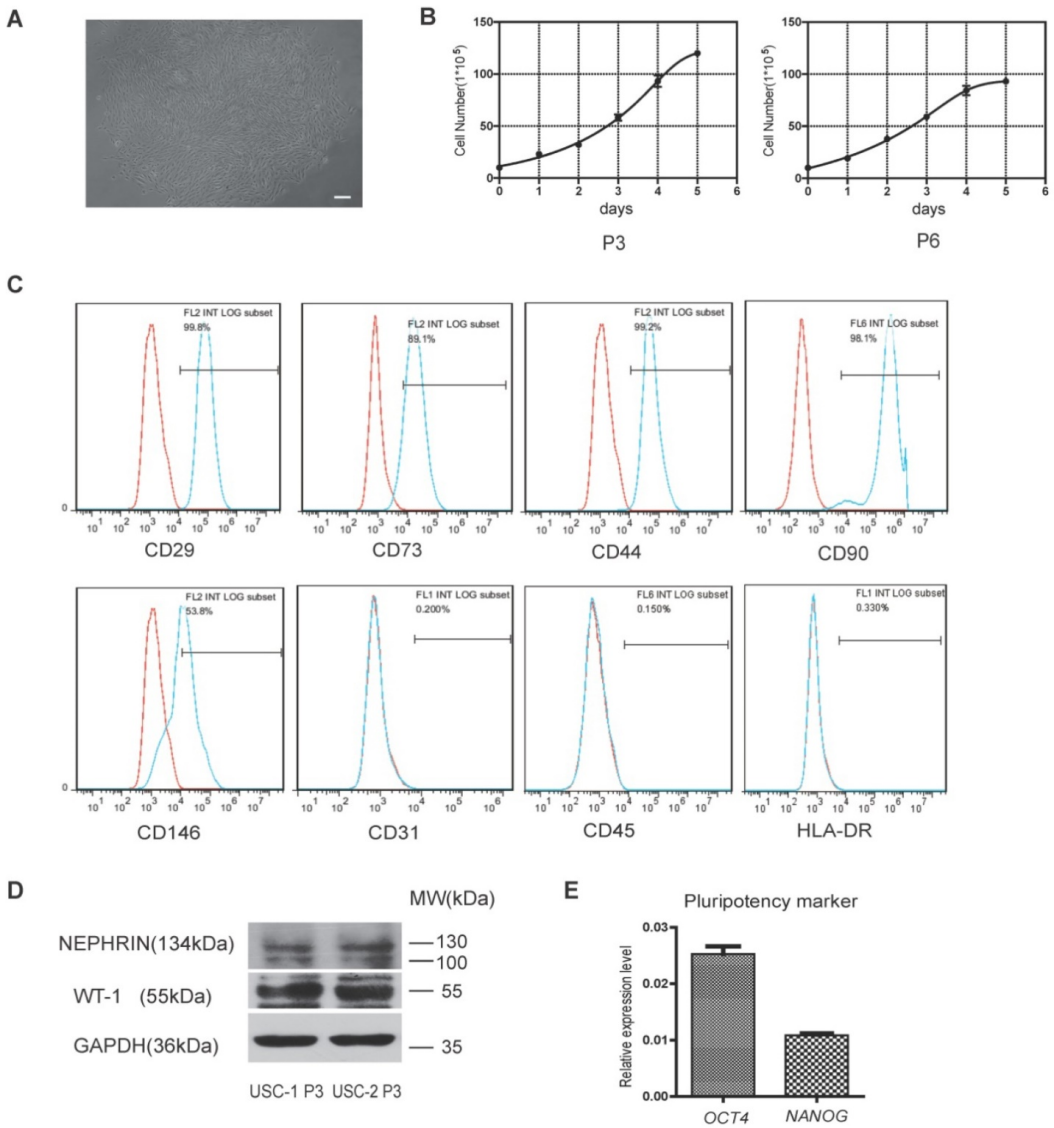
** Characterization of human urine-derived stem cells (USCs).** (**A**) Spindle-shaped morphology of USCs (scale bar = 50 µm). (**B**) Growth curves for USCs of different passages (P3 and P6). (**C**) After 3 passages, the majority of isolated USCs expressed MSC markers CD29, CD73, CD44, CD90, and CD146 but not CD31, C45, and HLA-DR. (**D**) Protein expression of renal markers Nephrin and WT-1 in USCs from two healthy people. (**E**) Relative expression levels of pluripotency markers *OCT4* and *NANOG* in USCs.

Cells isolated from human urine samples were cultured for 3-5 days, and tiny colonies of slender spindle-like cells were observed (Figure 1A). The cells proliferated rapidly *in vitro*, with cells of passages 3 and 6 (P3 and P6) exhibiting rapid proliferation capacity (Figure 1B). Flow cytometric analysis showed that USCs expressed common surface markers of MSCs, including CD29, CD73, CD44, CD90, and CD146, but not endothelial or hematopoietic cell surface markers, including CD31 and CD45. Low expression of human leukocyte antigen (HLA)-DR suggested the low immunogenicity of USCs (Figure 1C). Western blot analysis showed USCs expressed specific renal markers including nephrin and Wilms' tumor-1 (WT1; Figure 1D). qRT-PCR analysis showed that USCs expressed pluripotency markers, including OCT4 and NANOG (Figure 1E).

### USCs reduced renal tubular injury and cell apoptosis and inhibited local inflammation in the renal IRI model

**Figure 2:**
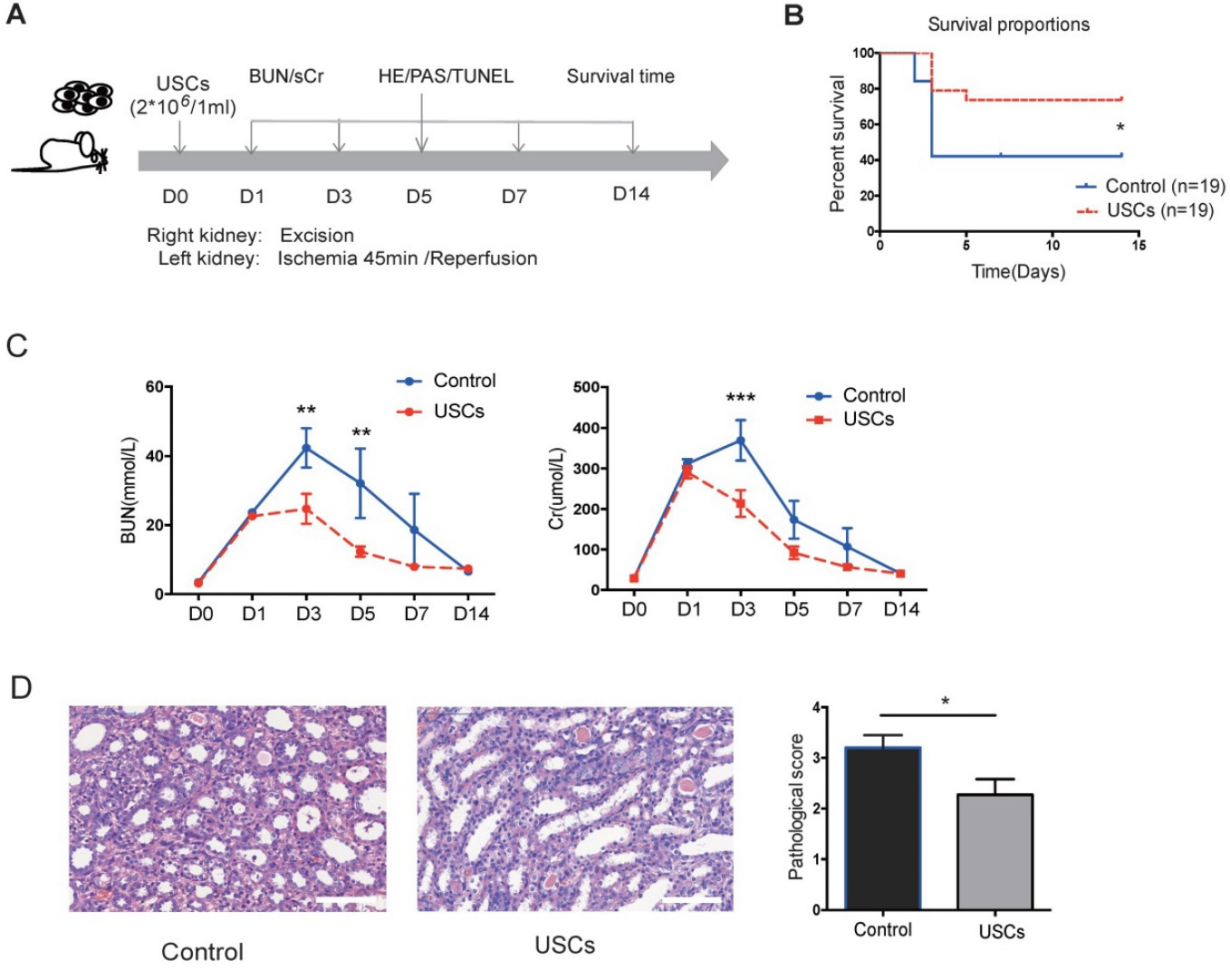
** The protective effects of USCs on renal function after renal IRI.** (**A**) Diagram of the ischemia-reperfusion injury (IRI) animal model. (**B**) Survival curves for the control and USC-treated groups (Control group, n=19; USC-treated group, n=19; *P*= 0.0328). (**C**) Time-dependent changes in blood urea nitrogen (BUN) and serum creatinine (sCr) in control (n=25) and USC-treated groups (USCs, n=26) at days 1, 3, 5, 7 and 14 after IRI (BUN: *P*= 0.0028 on day 3, *P*= 0.0066 on day 5; sCr: *P*<0.0001 on day 3). Data represent the mean ± SEM, *P<0.05, **P<0.01, ***P<0.001. (**D**) Histopathological scores for control (n=10) and USC-treated groups (USCs, n=11) on day 7 (*P*= 0.0311). Scale bars =100 µm. Data represent the mean ± SEM, *P<0.05, **P<0.01.

Compared to the control treatment, USC treatment significantly increased rat survival (*P* < 0.05), decreased sCr and BUN levels at day 3 post-injection (*P* < 0.05), and reduced the pathological score for renal injury at day 7 post-injection (*P* < 0.05; Figure 2B-D). These results indicated that intravenous transfusion of USCs ameliorated renal injury and protected renal function in the rat IRI model.

**Figure 3:**
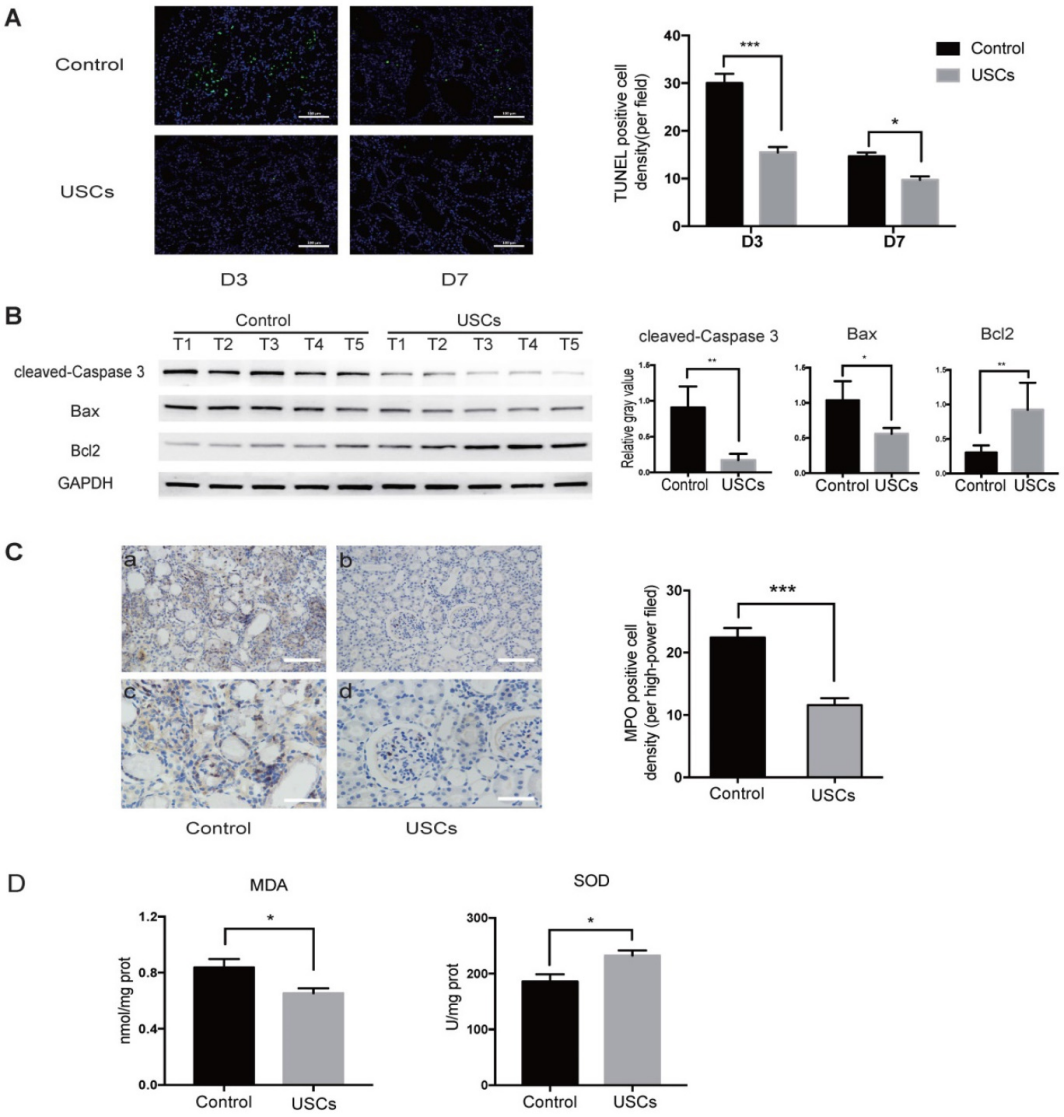
** USCs reduced the expression of apoptosis-related proteins, inflammatory cell infiltration, and oxidative stress level in the kidney after IRI.** (**A**) TUNEL staining analysis of the apoptosis among renal tubular epithelial cells in the control (n=5) and USC-treated group (USCs, n=5) on days 3 and 7 after IRI. Scale bars = 100 µm, *P*<0.001 on day 3, *P*= 0.0139 on day 7. (**B**) Western blot analysis of cleaved-caspase-3 and Bcl2/Bax expression in the control group (n=5) and USC-treated group (n=5) on day 3 after IRI. GAPDH was used as a loading control. (**C**) MPO staining analysis of neutrophil infiltration in the kidney tissue on day 3. n=5 in each group. Scale bars = 200 µm in a,b; 100 µm in c,d; *P*<0.001. (**D**) SOD and MDA analysis of oxidative stress in kidney tissue on day 3 after IRI. n=4 in each group. *P*= 0.0378 for MDA, *P*= 0.0262 for SOD. Data represent the mean ± SEM. *P<0.05, **P<0.01, ***P<0.001.

Compared to the control treatment, USC treatment also significantly decreased apoptosis in the IRI kidney at days 3 and 7 as indicated by TUNEL staining (Figure 3A). Western blot analysis showed that expression of pro-apoptotic cleaved-caspase-3 and Bax was decreased, whereas anti-apoptotic Bcl2 expression was increased in the kidney of USC-treated rats at day 3 (Figure 3B). MPO staining showed significantly fewer infiltrating neutrophils in the IRI kidney of hUSC-treated rats at day 3 (*P* < 0.001; Figure 3C). Additionally, the MDA content was significantly reduced (*P* < 0.05) and SOD activity was significantly increased (*P* < 0.05) at day 3 in the kidney of the USC-treated rats (Figure 3D). Taken together, these results suggest that USC treatment reduced cell apoptosis, neutrophil infiltration, and oxidative stress in the IRI kidney.

### Most injected hUSCs were trapped in lung tissue and not found in damaged kidney tissue

To test whether transfused USCs integrate into the injured tissue, we labeled USCs with PKH26 before injection into IRI rats through the dorsal vein of the penis and harvested rat organs, including the lungs, liver, spleen, and damaged kidneys, 24 h later. Fluorescence imaging showed that most of the injected hUSCs were trapped in lung tissue and did not reach the damaged kidney tissue (Figure S1A).

We also performed human nuclear antigen (hNA) staining of IRI kidney slices to distinguish human cells from rat tissues at day 7 post-injection. The results demonstrated that, while USCs were found in the kidney after infusion into the renal artery after IRI (positive control); few USCs had integrated into the tubular epithelial lining of the kidney after injection via the dorsal vein of the penis in rats (Figure S1B). This result supported the idea that direct contact between injected USCs and injured renal cells might not be required the protective action of the injected USCs.

### USC-Exo protected against IRI *in vivo* and inhibited oxidative stress in HK-2 cells after H/R injury *in vitro*

We collected CM from USCs to test whether USC-secreted soluble factors affect renal tubular cell injury *in vitro*. H/R injury in normal human kidney cortex/proximal tubule cells HK2 was induced by incubation in glucose- and serum-free medium in 1% O_2_ for 48 h followed by reoxygenation with normal O_2_ in either USC CM or control medium for 6 h (Figure S2A). The SOD activity and MDA content were analyzed to determine the level of oxidative stress in the injured cells. The results showed that USC CM reduced oxidative stress by increasing SOD activity and decreasing MDA content (Figure S2B).

**Figure 4:**
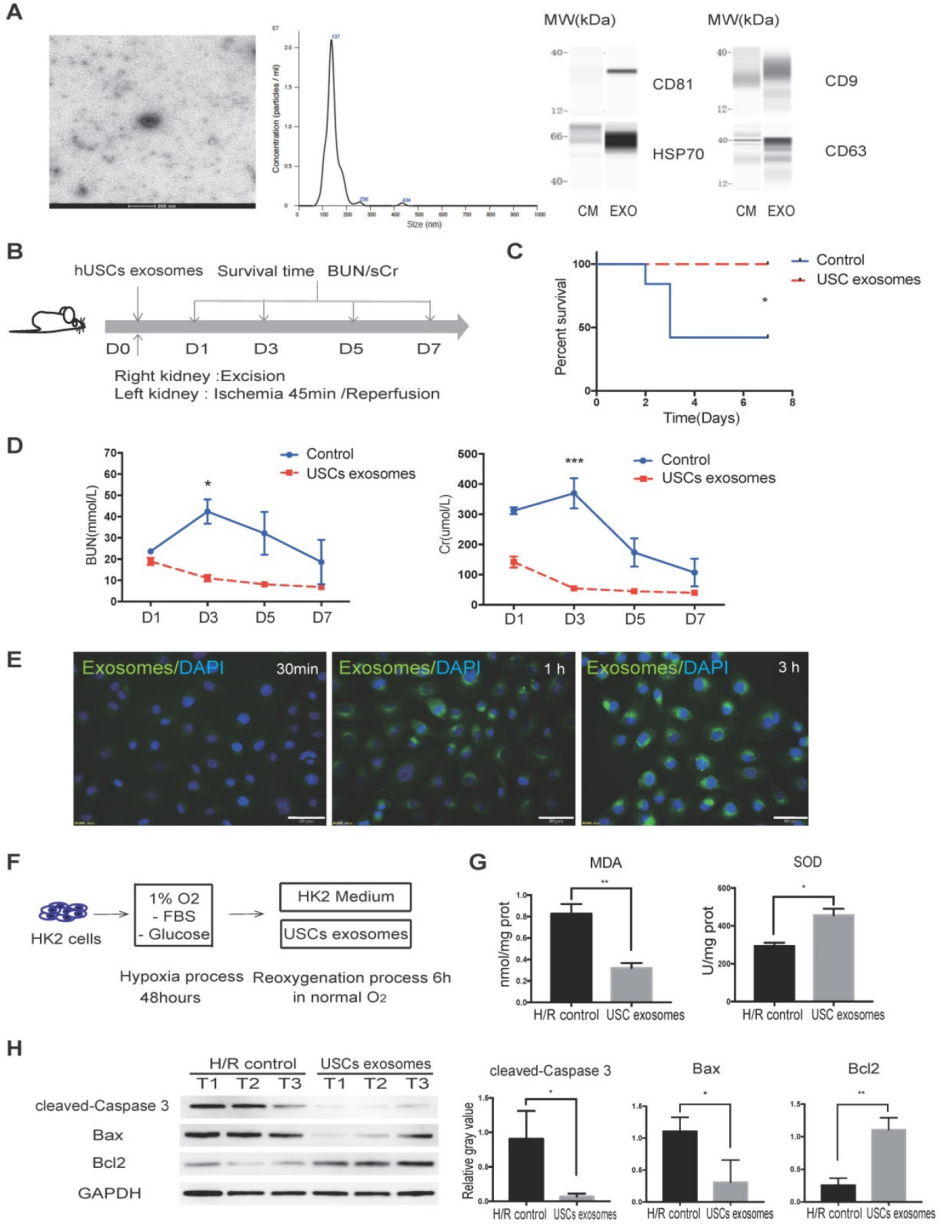
** USC-Exo protect renal function *in vivo* and HK2 cells after H/R injury *in vitro*.** (**A**) Characterization of USC-Exo. TEM images of exosomes isolated from USC CM (scale bar = 200 nm). Size analysis of USC-Exo (mean diameter = 144.9 ± 41.6 nm). Western blot analysis of exosomal markers CD81, CD9 CD63, and HSP70. CM served as the control. (**B**) Diagram of IRI animal model with exosome treatment. (**C**) Survival curve analysis for the two groups (control group, n=19; USC-Exo-treated group, n=6; *P*=0.0201). (**D**) Time-dependent changes in BUN and sCr in the control group (n=25) and USC-Exo-treated group (USC-Exo, n=6) on days 1, 3, 5, and 7 after IRI (BUN: *P*= 0.0228 on day 3; sCr: *P*= 0.0589 on day 1,* P*<0.001 on day 3). (**E**) After incubation of HK2 cells with USC-Exo (PKH67, green fluorescence) for 30 min, 1 h, or 3 h, and washing with PBS, the fusion of exosomes and cells was observed fluorescence microscopy (scale bars = 50 µm). (**F**) Schematic diagram of H/R-induced injury in HK2 cells with or without USC-Exo treatment during the reoxygenation process. (**G**) Oxidative stress levels in HK2 cells after H/R in the two groups were determined by measuring MDA and SOD (*P*=0.0073 for MDA, *P*=0.0132 for SOD). Each experiment was repeated three times. (**H**) Protein expression of cleaved-Caspase-3 and BCL2/BAX in the indicated groups. After USC-Exo treatment, the expression levels of cleaved-Caspase-3 and BAX in HK2 cells were decreased significantly. Each experiment was repeated three times. GAPDH was used as loading control. Data represent the mean ± SEM. *P<0.05, **P<0.01, ***P<0.001.

Previous studies reported that exosomal contents secreted from MSCs are protective against IR injury 31, 32. We, therefore, aimed to investigate whether USCs protect HK2 cells from H/R-induced injury via secreted exosomal factors. Following the latest guidelines for exosome isolation and characterization 33, we isolated USC-secreted EVs and examined them by TEM. We found that the USC-EVs showed a sphere-like morphology with diameters of 100-150 nm. We further carried out particle size analysis on the isolated exosomes and measured a mean diameter of 144.9 ± 41.6 nm. The exosomes were found to express exosomal markers, including CD81, CD9, CD63, and HSP70, by western blot analysis (Figure 4A). These results demonstrated the exosomal identify of the EVs isolated from USCs.

To further evaluate the protective effect of USC-Exo against renal IR injury, we again used the lethal rat renal IRI model and injected USC-Exo into the IRI rat at the time of reperfusion (Figure 4B). We found that the levels of BUN and sCr in the middle-dose group (20 μg/rat) were similar to those in the high-dose group (40 μg/rat) and lower than those in the low-dose group (10 μg/rat; data not shown). Therefore, in the subsequent experiments, we used the middle dose (20 μg/rat) of USC-Exo.

We found that the rats in the USC-Exo treated group did not die during the observation period (Figure 4C). We continuously monitored the changes in BUN and sCr levels and found that these levels in the USC-Exo treated group were significantly lower than those in the control group (Figure 4D). These results demonstrated that treatment with USC-Exo could also ameliorate renal damage, similarly to USCs, in the rat IRI model.

We further examined the protective effect of USC-Exo *in vitro*. To demonstrate the fusion of exosomes with the target cells, we labeled USC-Exo with green fluorescent dye (PKH67) and incubated DAPI-stained HK2 cells with the labeled exosomes for 30 min, 1 h, or 3 h. After these different periods, we washed the cells with PBS and detected the fluorescence signals among the plated cells. We found that the intracellular fluorescence signal gradually increased with the extension of the incubation time. After incubation for 3 h, the fluorescence signal of USC-Exo could be detected in most cells (Figure 4E). After exposure of the cells to hypoxia for 48 h, we added 100 μL USC-Exo solution (20 μg/mL) into the HK2 medium during the re-oxygenation process for 6 h (Figure 4F). The MDA content was significantly lower (*P* < 0.05) and SOD activity significantly greater (*P* < 0.05) in the USC-Exo-treated group compared with the control group (Figure 4G). Western blot analysis showed that treatment with USC-Exo reduced H/R-induced expression of cleaved caspase-3 and BAX, but increased BCL2 expression (Figure 4H). These results demonstrate the protective effect of USC-Exo against H/R injury in HK2 cells.

### *miR-146a-5p* in USC-Exo inhibited IRAK1 expression

**Figure 5:**
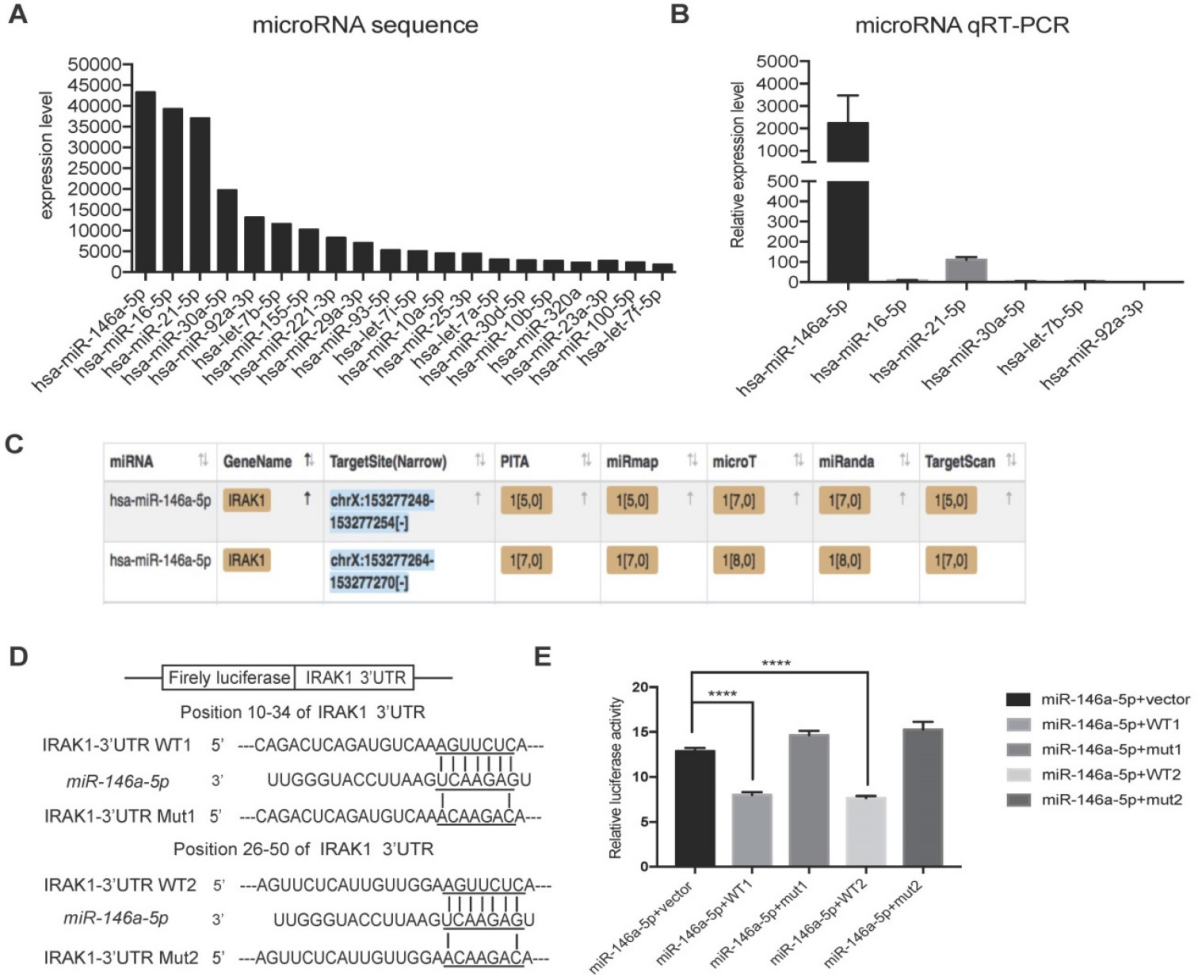
** miRNA sequencing of USC-Exo contents and potential targets of *miR-146a-5p.***(**A**) The top 20 most-enriched miRNAs in USCs-Exo. (**B**) qRT-PCR analysis of the expression levels of the top 6 most-enriched miRNAs in USCs-Exo. (**C**) StarBase analysis of the target genes of *miR-146a-5p*. (**D**) Sequence alignments of *miR-146a-5p* and its two candidate target sites in the 3'UTR of *IRAK1*. (**E**) Luciferase reporter assay of *miR-146a-5p* mimic-treated HEK293T cells, which overexpressed either IRAK1-wildtype 3'UTR (WT1 and WT2) or IRAK1-mutant 3'UTR (mut1 and mut2). Data represent the mean ± SEM. *P<0.05, **P<0.01, ***P<0.001.

To further investigate the mechanism responsible for the protective effect of USC-Exo, we hypothesized that miRNAs might play important roles based on reports that exosomal miRNAs are involved in multiple physiological and pathological processes 34. RNA sequencing analysis revealed that 20 annotated miRNAs were enriched in the USC-Exo (Figure 5A). We next confirmed the expression levels of the top six enriched miRNAs by qRT-PCR and identified *miR-146a-5p* as the most highly expressed miRNA in USC-Exo (Figure 5B). To further determine the functions of *miR-146a-5p*, we searched the miRNA target prediction analysis database Starbase v2.0 (http://starbase.sysu.edu.cn) and found 451 genes as potential targets of *miR-146a-5p*. *Interleukin-1 receptor-associated kinase 1 (IRAK1)* was ranked highest among the predicted targets of *miR-146a-5p*, with two predicted target sites for *miR-146a-5p* in the 3' untranslated region (UTR) of the transcript of *IRAK1* (Figure 5C). In order to verify the direct binding of *miR-146a-5p* to the 3'UTR region of *IRAK1* gene, we then cloned the wild-type and mutant 3'UTR of *IRAK1* downstream of a firefly luciferase cassette in a luciferase reporter vector. Co-transfection of *miR-146a-5p* mimic with the wild-type reporter plasmids in HEK293T cells significantly reduced the luciferase activity, which was significantly reversed by co-transfection with two mutant reporter plasmids (Figure 5D, E). These results indicated that *miR-146a-5p* from USC-Exo may bind the *IRAK1* mRNA 3'UTR region and thereby inhibit IRAK1 expression via post-translational repression.

### USCs enhanced *miR-146a-5p* expression and inhibited IRAK1 expression and nuclear translocation of the NF-κB p65 subunit *in vivo* and *in vitro*

**Figure 6:**
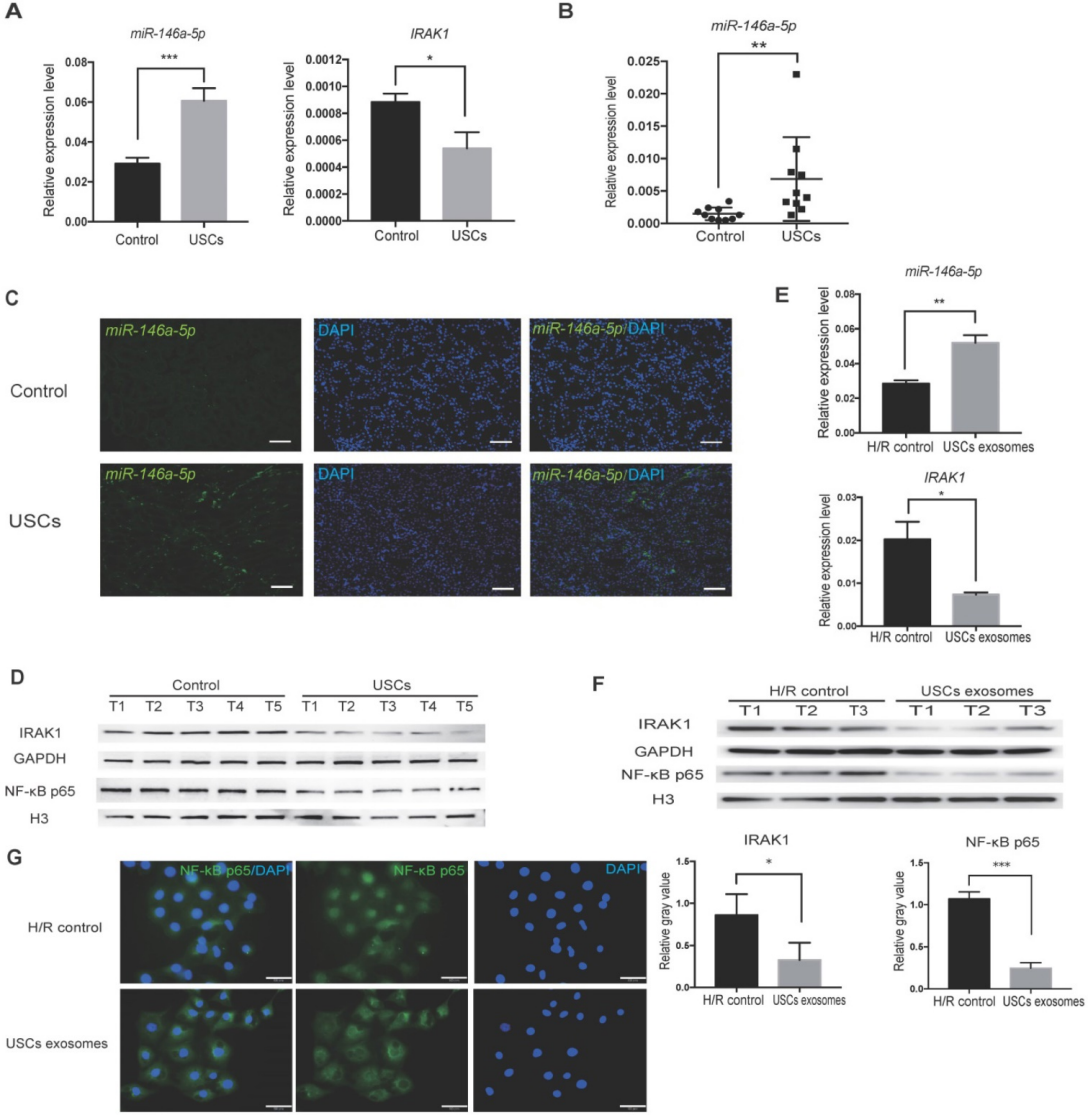
** USCs or USC-Exo upregulate *miR-146a-5p* expression, which targets the IRAK1 and NF-κB signaling *in vivo* and *in vitro*.** Rat IRI was induced before treatment with or without USCs (annotated as USCs and control, respectively). (**A**) qRT-PCR analysis of the relative expression levels of *miR-146a-5p* (*P*=0.0004) and* IRAK1* (*P*=0.0227). (**B**) qRT-PCR analysis of the relative expression levels of *miR-146a-5p* in rat serum exosomes on day 3 (n=10 in each group, *P*=0.0012). (**C**) FISH images of *miR-146a-5p* expression in kidney sections (scale bars = 100 µm). (**D**) Western blot analysis of the protein levels of IRAK1 and nuclear NF-κB p65. n=5 in each group. GAPDH and H3 were used as loading controls, respectively. H/R injury was induced in HK2 cells in the absence or presence of USC-Exo (annotated as HK2 medium and HK2 medium+exosomes, respectively). (**E**) qRT-PCR analysis of the relative expression levels of *miR-146a-5p* (*P*=0.0078) and *IRAK1* (*P*=0.0358). (**F**) Western blot assay of the protein levels of IRAK1 and nuclear NF-κB p65. n=3 in each group. GAPDH and H3 were used as loading controls, respectively. (**G**) Immunofluorescence analysis showed that NF-κB p65 in HK2 cells was transferred from the cytoplasm to the nucleus after H/R treatment, and this nuclear translocation of NF-κB p65 could be inhibited by USC-Exo treatment (scale bars = 50 µm). Each experiment was repeated three times. Data represent the mean ± SEM. *P<0.05, **P<0.01.

To further confirm the hypothesis that *miR-146a-5p* is critical to the protective effect of USCs in IRI, we first identified the expression level of *miR-146a-5p* in the kidney tissue of USC-treated IRI rats. qRT-PCR analysis revealed upregulated *miR-146a-5p* expression in the kidney of the USC-treated IRI group (Figure 6A). To further determine whether USC-secreted *miR-146a-5p* reaches the damaged kidney tissue via exosomal delivery, we collected serum samples from the rats on day 3 after USC treatment, extracted the exosomes, and detected the expression level of *miR-146a-5p*. The results showed greater expression of exosomal *miR-146a-5p* in the serum of the USC-treated group compared with the control group (Figure 6B). FISH images further demonstrated the increased expression of *miR-146a-5p* in damaged kidney tissue (Figure 6C). The mRNA (Figure 6A) and protein (Figure 6D) expression levels of IRAK1 were further examined and shown to be downregulated in the kidney of USC-treated IRI rats.

A corresponding *in vitro* experiment was conducted using H/R-induced injury in HK2 cells. Treatment with USC-Exo significantly upregulated *miR-146a-5p* expression and downregulated *IRAK1* expression in these cells (Figure 6E). In agreement with the *in vivo* observations, western blotting showed that IRAK1 protein expression as well as nuclear translocation of the NF-κB p65 subunit was reduced by treatment with USC-Exo (Figure 6F). We further performed staining of NF-κB p65 in the USC-Exo treated cells and found that in the control HK2 cells after H/R, NF-κB p65 was localized in the nucleus. In contrast, in the USC-Exo treated HK2 cells after H/R, some NF-κB p65 remained in the cytoplasm, and its nuclear localization level was significantly reduced (Figure 6G). These results suggested that the protective effect of USCs against renal injury may occur via exosomal *miR-146a-5p-*mediated downregulation of NF-κB signaling.

### *miR-146a-5p* inhibited IRAK1 expression and nuclear translocation of NF-κB p65 in response to H/R in HK2 cells

**Figure 7:**
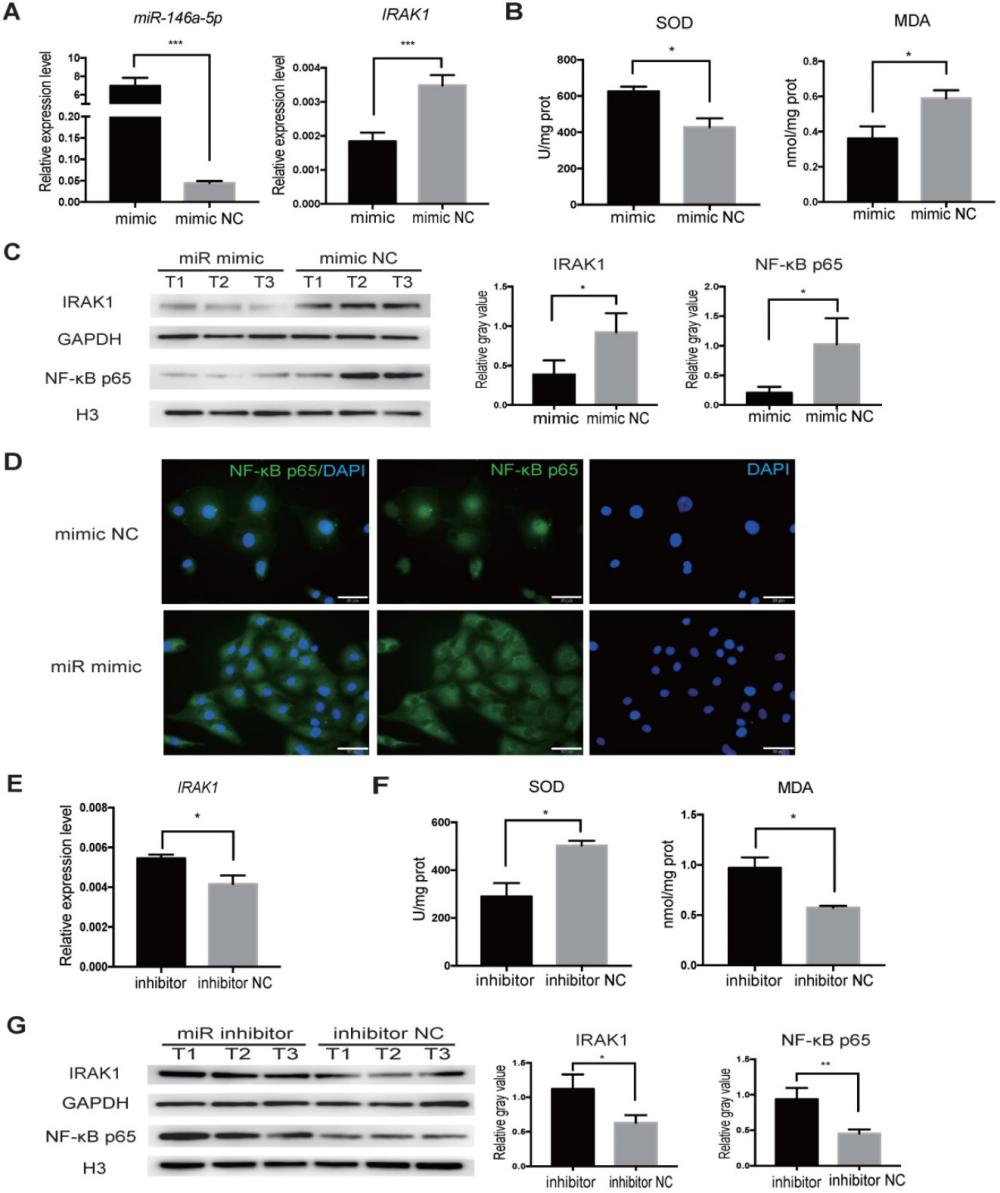
***miR-146a-5p* mimic reduces oxidative stress and inhibits IRAK1 and NF-κB signaling in H/R-induced injury of HK2 cells.** HK2 cells were transfected with *miR-146a-5p* mimic or its inhibitor before the H/R process. (**A**) qRT-PCR analysis of the relative expression levels of *miR-146a-5p* and *IRAK1* in the mimic and mimic negative control (mimic NC) groups (*P*<0.001). (**B**) Analysis of SOD activity and MDA content in the mimic and mimic NC groups after H/R (*P*=0.0216 for SOD, *P*=0.0497 for MDA). (**C**) Western blot analysis of the protein levels of IRAK1 and nuclear translocation of NF-κB p65 in the mimic and mimic NC groups. n=3 in each group. GAPDH and H3 were used as loading controls, respectively. (**D**) Immunofluorescence analysis of the nuclear translocation of NF-κB p65 in the mimic and mimic NC groups after H/R treatment (scale bars=50 µm). (**E**) qRT-PCR analysis of the relative expression levels of *IRAK1* in the inhibitor and inhibitor negative control (NC) groups (*P*=0.015). (**F**) Analysis of SOD activity and MDA content in the inhibitor and inhibitor NC groups after H/R (*P*=0.0228 for SOD, *P*=0.0192 for MDA). (**G**) Western blot analysis of the protein levels of IRAK1 and nuclear translocation of NF-κB p65 in the inhibitor and inhibitor NC groups. n=3 in each group. GAPDH and H3 were used as loading controls, respectively. Each experiment was repeated three times. Data represent the mean ± SEM. *P<0.05, **P<0.01, ***P<0.001.

We next aimed to delineate the regulatory relationship between *miR-146a-5p* and IRAK1 using the H/R-induced injury model *in vitro*. We transfected HK2 cells with *miR-146a-5p* mimic, and increased *miR-146a-5p* expression was confirmed by qRT-PCR (Figure 7A). The transfected cells were subjected to H/R injury at the same time, and qRT-PCR analysis showed that *miR-146a-5p* mimic resulted in a significant decrease in *IRAK1* mRNA expression (Figure 7A). Furthermore, transfection with the *miR-146a-5p* mimic could reduce oxidative stress, increase SOD activity, and decrease MDA content (Figure 7B). Western blot analysis showed that transfection with the *miR-146a-5p* mimic resulted in a significant decrease in IRAK1 protein expression and NF-κB p65 subunit nuclear translocation (Figure 7C). The results of immunofluorescence staining also showed that the *miR-146a-5p* mimic could reduce the nuclear localization of NF-κB p65 (Figure 7D). We also treated HK2 cells with a miRNA inhibitor and found that the expression level of IRAK1 in the inhibitor-treated group was higher than that in control group (Figure 7E). *miR-146a-5p* inhibitor could aggravate the oxidative stress, decrease SOD activity, and increase MDA content (Figure 7F). Western blot analysis also showed that *miR-146a-5p* inhibitor could support IRAK1 protein expression and NF-κB p65 subunit nuclear translocation (Figure 7G).

Together these results indicated the indispensable role of *miR-146a-5p* in regulating IRAK1 expression and NF-κB p65 subunit nuclear translocation in H/R-induced injury.

### Knock down of IRAK1 inhibited the nuclear translocation of NF-κB p65 in response to H/R in HK2 cells

To further test whether IRAK1 could regulate the nuclear translocation of NF-κB p65, we knocked down IRAK1 expression by siRNA. Among three target siRNAs (target sequence 1: GGACATCCTTCTGGGTACA, target sequence 2: GAGCCACCGCAGATTATCA, target sequence 3: GCCCGAAGAAAGTGATGAA), we found that si-2 and si-3 had the higher inhibition efficiencies (Figure S3A), and we choose to use si-2 and -3 in combination (Figure S3B). Western blot analysis showed that the nuclear level of NF-κB p65 was reduced upon knock down of IRAK1 (Figure S3C). These results indicate that knock down of IRAK1 inhibited the nuclear translocation of NF-κB p65 in HK2 cells after H/R injury.

## Discussion

In this study, we demonstrated the renal protective effect of USCs *in vitro* and *in vivo*. In a rat renal IRI model, USC therapy significantly reduced mortality, decreased sCr and BUN levels, and attenuated renal tubular injury. We further showed that USCs possessed anti-inflammatory, anti-oxidative, and anti-apoptotic effects, which are known to contribute to the protective effects of other types of stem cells against renal IRI 35. Most importantly, we revealed a novel mechanism of USC function: USC-Exo delivery of *miR-146a-5p* to downregulate *IRAK1* expression and subsequently inhibit NF-κB signaling.

The potential therapeutic efficacy of MSCs in kidney diseases has been widely reported 11, 36. Due to the invasive collection process and limited proliferation abilities of MSCs, we aimed to explore the utility of USCs in renal IR injury repair. USCs expressed stem cell markers such as SSEA4, Sox2, and Oct3/4 at the early stage of isolation. USCs stably expressed MSC-specific surface molecules (CD29, CD44, CD73, CD90, etc.) and did not express hematopoietic stem cell markers (CD133, CD34, CD45, etc.) or endothelial cell markers (CD31, etc.), suggesting that USCs are not hematopoietic stem cells or endothelial precursor cells, but a special type of MSCs. A previous study found that most USCs expressed cytokeratin 7 (CK7), suggesting that these USCs may be uroplakin basal cells. However, the same group also found that USCs expressed renal markers (CD224, CD13, NR3C2, Pax2, and Pax8) and renal epithelial cell-specific markers (CD146 and podocin), but did not express markers of renal tubular epithelial cells, ureteral epithelial cells, or urethral epithelial cell markers. This suggested that USCs may be derived from the junction of visceral and parietal epithelial cells of the renal glomerulus, rather than urethral epithelial basal cells 13, 37. However, the real source of USCs remains to be explored. In our experiments, the surface molecular expression patterns of the USCs were consistent with those described previously. The major advantages of USCs include the non-invasive collection process and non-costly isolation/expansion protocol. In agreement, Tian et al. 21 and we reported that USCs exert anti-inflammatory and anti-apoptotic effects to facilitate the repair of ischemic acute kidney injury in rats. Tian et al. detected USCs in tubular epithelium, because they injected USCs directly into the upper, middle and lower cortex of the kidney. We, however, did not observe the incorporation of USCs into the renal tissue after intravenous administration. This difference may be related to the different injection routes employed by the studies. A recent study also concluded that hUSCs have a nephron-protective effect on renal function via anti-inflammatory, anti-oxidative stress, and anti-fibrotic activities in a rat model of chronic kidney disease, which was induced by IRI and gentamicin administration 38. Another recent study also described the therapeutic effects of USCs in a rat model of cisplatin-induced AKI 39. Both of these studies together with our study support the effectiveness of USCs in the treatment of renal-related diseases.

Thus, our present study also aimed to explore the indirect mechanism by which USCs protect against IRI, given that we did not found evidence of direct-targeted migration of USCs into the damaged tissue. The effects of MSCs are primarily mediated via complex paracrine actions and the transfer of EVs/exosomes packaging various molecules, such as bioactive lipids, proteins, and RNAs 40. MSC-derived EVs could restore renal structure and function via their immunomodulatory, anti-apoptosis, and proliferation stimulation abilities in AKI models 41-44. We, therefore, investigated the role of EVs/exosomes in USC therapy for renal IRI. USC-Exo were isolated from USC CM and identified as exosomes based on size and the expression of exosome-specific markers. Our results showed that USC-Exo could preserve renal function after IRI, improve the survival rate of IRI rats, and reduce the levels of sCr and BUN *in vivo*. Consistently, USC-Exo also protected HK2 cells from H/R injury and reduced oxidative stress *in vitro*.

One component of exosomes that is found at extremely high levels in miRNA. Exosomal miRNAs have been implicated in many processes such as cartilage repair, renal fibrosis inhibition, and IRI in myocardium cells 45-47. Thus, we performed miRNA sequencing of the contents of USC-Exo and identified *miR-146a-5p* as the most enriched miRNA. The role of *miR-146a-5p* has been reported in renal diseases, and treatment with *miR-146a-5p* was shown to inhibit renal fibrosis *in vivo* 48. *miR-146a-5p* expression in the kidneys as well as its urinary excretion are specifically associated with the development of interstitial lesions and correlated with inflammatory cell infiltration 49. *miR-146a*(-/-) mice exhibit more extensive tubular injury, inflammatory infiltrates, and fibrosis than wild-type mice 50*. miR-146a-5p* expression is decreased in the glomeruli of patients with type 2 diabetes, which correlates with increased albuminuria and glomerular damage 51. One study also found that the expression levels of nine miRNAs (*miR-21, miR-20a, miR-146a, miR-199a-3p, miR-214, miR-192, miR-187, miR-805, and miR-194*) were altered in kidney tissues of C57BL/6 mice exposed to unilateral thermal warm ischemia compared with their levels in the control group. Among these miRNAs, *miR-146a* showed significantly increased expression at 14 days after IRI 52. In our experiment, the time point chosen for *miR-146a* detection was before day 7 after IRI. Therefore, the expression level of *miR-146a* in the IRI control group was still at a low level, while the expression level of *miR-146a* in the USC-treated group was significantly increased. These results suggest a protective role of *miR-146a-5p* against renal injury in both mice and human.

We further investigated the downstream mechanism of USC-Exo derived *miR-146a-5p* and showed that it could downregulate the expression of IRAK1. As a serine-threonine kinase, IRAK1 mediates the Toll-like receptor (TLR) and interleukin-1 (IL-1) signaling pathways and therefore plays a critical role in innate immunity 53. *miR-146a*-mediated downregulation of IRAK1 is involved in a wide spectrum of diseases, including autoimmune disorders, cancer, diabetes, or neuropathic pain 54-57, as well as in cerebral, hepatic, or intestinal IRI 58-60. Considering research showing that downregulation or inhibition of IRAK1 can attenuate high glucose- or lipopolysaccharide (LPS)-induced renal injury (podocyte apoptosis) 61, 62, in combination with our current results, a critical role for IRAK1 in renal IRI is strongly supported.

We also showed that *miR-146a-5p* is implicated in USC-Exo suppression of IRAK1 expression and NF-κB signaling. *miR-146a-5p* was also reported to be an NF-κB dependent gene that can directly downregulate the production of pro-inflammatory cytokines, in control of TLR and cytokine signaling through a negative feedback regulation loop involving down-regulation of tumor necrosis factor (TNF) receptor-associated factor 6 (TRAF6) and IRAK1 protein levels 63, 64. Previous studies have demonstrated that *miR-146a*-deficient mice develop severe gouty arthritis via TRAF6, IRAK1 and NALP3 (NACHT, LRR and PYD domain-containing protein 3)-induced inflammasome dysregulation 65. Inhibition of chemokine signaling prevents the development of inflammation and fibrosis after IRI in *miR-146a*(-/-) mice 50. Our experiments also showed that *miR-146a-5p* regulates downstream IRAK1 and NF-κB signaling. Taken together, our results indicate that the role of *miR-146a-5p* in limiting inflammation in renal IRI may occur though IRAK1/NF-κB signaling.

Some limitations of our study should be considered. The most recognized gold standard for separating exosomes is "differential ultracentrifugation", in which cells, debris, and large-diameter vesicles are removed step-by-step by differential centrifugation, and exosomes are precipitated and enriched by ultracentrifugation. In this study, a more convenient and easy-to-operate commercial kit was utilized for exosome extraction, which may have affected the purity of the isolated exosomes. Also, if USCs can be treated by gene editing, key factors of the exosome secretion pathway can be knocked out or inhibited, thereby blocking exosome secretion. USCs that lose the ability to secrete exosomes can be injected into animal models, and investigations of the changes in the renal protection ability of such cells can further prove the important role of exosomes in the renal protective effects of USCs. Finally, due to the limitations of our experimental conditions, it was impossible to trace and detect the exosomes released by USCs *in vivo*. If USC-secreted exosomes could be specifically labeled and directionally tracked *in vivo*, the enrichment of exosomes in the kidney instead of whole cells could be detected. This design could more directly prove that USCs offer renal protection through exosomes.

## Conclusions

In summary, we demonstrated that USCs protect against renal injury in a lethal rat IRI model via the interplay of anti-oxidative, anti-inflammatory, and anti-apoptotic cytoprotective effects. Furthermore, using an H/R-induced oxidative stress cell model *in vitro*, we identified the key player, *miR-146a-5p* in the USC-Exo, which mediates the cytoprotective effects by downregulating IRAK1/NF-κB signaling. These findings provide a theoretical foundation for the possibility of using the non-invasive and cost-effective USCs or USC-Exo for the treatment of AKI.

## Supplementary Material

Supplementary figures.

## Figures and Tables

**Table 1:** Antibodies used in this study

Protein name	Manufacturer	Catalog No.
Rabbit monoclonal anti-WT1 antibody	Abcam	89901
Rabbit polyclonal anti-Nephrin antibody	Abcam	72908
Mouse Anti-Human CD29	BD	555443
Mouse Anti-Human CD31	BD	560984
Mouse Anti-Human CD34	BD	560940
Mouse Anti-Human CD44	BD	550989
Mouse Anti-Human CD45	BD	340943
Mouse Anti-Human CD73	BD	550257
Mouse Anti-Human CD90	BD	561971
Mouse Anti-Human CD146	BD	550315
Mouse Anti-Human HLA-DR antibody [MEM-12]	Abcam	28323
Anti-cleaved Caspase-3 antibody	Abcam	49822
Bcl-2 (D55G8) Rabbit mAb (human specific)	Cell Signaling	4223
Bcl-2 Polyclonal Antibody (for rat)	Invitrogen	PA5-27094
Bax Rabbit Antibody	Cell Signaling	2772
ExoAb Antibody Kit (CD9, CD63, CD81, HSP70 antibodies, rabbit anti-human)	SBI	#EXOAB-KIT-1
Anti-Human Nuclear Antigen antibody [235-1]	Abcam	191181
IRAK1 Polyclonal Antibody	Invitrogen	PA5-19855
NF-κB p65 (D14E12) XP® Rabbit mAb	Cell Signaling	8242S

**Table 2:** Primers used for RT-qPCR analysis

Gene name	Forward sequence (5′-3′)	Reverse sequence (5′-3′)
**Human gene**		
*h-ACTIN*	CACCCAGCACAATGAAGATCAAGAT	CACCCAGCACAATGAAGATCAAGAT
*h-OCT4*	GAGTGAGAGGCAACCTGGAGAAT	ACCGAGGAGTACAGTGCAGTGAA
*h-NANOG*	GAGAAGAGTGTCGCAAAAAAGGA	TGAGGTTCAGGATGTTGGAGAGT
*h-IRAK1*	CGGTGCCAGGACCAAGTATCT	CCTCTCGTACACCTGGGTCATAG
*hsa-miR-146a-5p*	ACACTCCAGCTGGGTGAGAACTGAATTCCA	TGGTGTCGTGGAGTCG
*hsa-miR-16-5p*	ACACTCCAGCTGGGTAGCAGCACGTAAATA	TGGTGTCGTGGAGTCG
*hsa-miR-21-5p*	ACACTCCAGCTGGGTAGCTTATCAGACTGA	TGGTGTCGTGGAGTCG
*hsa-miR-30a-5p*	ACACTCCAGCTGGGTGTAAACATCCTCGAC	TGGTGTCGTGGAGTCG
*hsa-let-7b-5p*	ACACTCCAGCTGGGTGAGGTAGTAGGTTGT	TGGTGTCGTGGAGTCG
*hsa-miR-92a-3p*	ACACTCCAGCTGGGTATTGCACTTGTCCCG	TGGTGTCGTGGAGTCG
*U6*	CTCGCTTCGGCAGCACA	AACGCTTCACGAATTTGCGT
**Rat gene**		
*R-Gapdh*	TTCCTACCCCCAATGTATCCG	CATGAGGTCCACCACCCTGTT
*R-Irak1*	GACTTTGGTCTGGCTCGTTTCA	TCACTCCCACCTCTTCAGCCT
*rno-miR-146a-5p*	ACACTCCAGCTGGGTGAGAACTGAATTCCA	TGGTGTCGTGGAGTCG
*U6*	CTCGCTTCGGCAGCACA	AACGCTTCACGAATTTGCGT

## Abbreviations

AKI: acute kidney injury
BUN: blood urea nitrogen
DMEM: dulbecco's modified eagle's medium
HK2: human kidney cortex/proximal tubule cells
H/R: hypoxia-reoxygenation
USCs: urine-derived stem cells
USC-Exo: exosomes derived from human urine-derived stem cells
*IRAK1*: *interleukin-1 receptor-associated kinase 1*
IRI: ischemia reperfusion injury
miRNAs: microRNAs
NEAA: non-essential amino acid
*NF-κB*: *nuclear factor kappa-light-chain-enhancer of activated B cells*
qRT-PCR: quantitative reverse-transcriptase polymerase chain reaction
sCr: serum creatinine
SOD: superoxide dismutase activity
MDA: malonaldehyde
TUNEL: terminal-deoxynucleoitidyl transferase-mediated nick end labeling
