# Supplementary material for: Human urine-derived stem cells protect against renal ischemia/reperfusion injury in a rat model via exosomal miR-146a-5p which targets IRAK1
Source: Theranostics. 2020 Jul 25;10(21):9561–78. doi: 10.7150/thno.42153 (PMC7449916; doi:10.7150/thno.42153)
Supplement: Supplementary file 1 — Supplementary figures. [file thnov10p9561s1.pdf]

Figure S1. Low or no integration of USCs into the IR-injured kidney tissue.

(A) At 24 h after PKH26-positive USC treatment, immunofluorescence staining showed that PKH26-positive cells were mostly trapped in the lungs. A few positive cells were distributed in the liver, but no injected cells were found in the spleen or damaged kidney. Scale bars = 50 μm. (B) Immunohistochemical staining of human nuclear antigen (hNA) in kidney sections on day 7 after injection of USCs via the renal artery (positive control, red arrows) or dorsal vein of the penis. Scale bars = 200 μm in a,b; 100 μm in c,d.

Figure S2. Expression levels of *miR-146a-5p* and *IRAK1* in H/R-induced oxidative stress model after treatment with different types of medium.

(A) Schematic diagram of H/R-induced injury in HK2 cells treated with HK2 medium, fresh USC medium, or USC conditioned medium (CM). (B) SOD and MDA analysis in H/R-stimulated HK2 cells treated with or without USC CM (P=0.049 for MDA, P=0.0017 for SOD). Each experiment was repeated three times. Data represent the mean ± SEM. *P<0.05, **P<0.01, ***P<0.001.

Figure S3. Nuclear translocation of NF-κB p65 after *IRAK1* knock down in HK2 cells subjected to H/R injury in vitro.

(A) Western blot analysis of the protein levels of IRAK1 after treatment with three different siRNAs. GAPDH was used as the loading control. (B) Western blot analysis of the protein levels of IRAK1 in cells treated with siRNA(2+3). GAPDH was used as the loading control. (C) Western blot analysis of the nuclear translocation of NF-κB p65 in siIRAK1-treated and control cells. GAPDH and H3 were used as the loading controls, respectively.

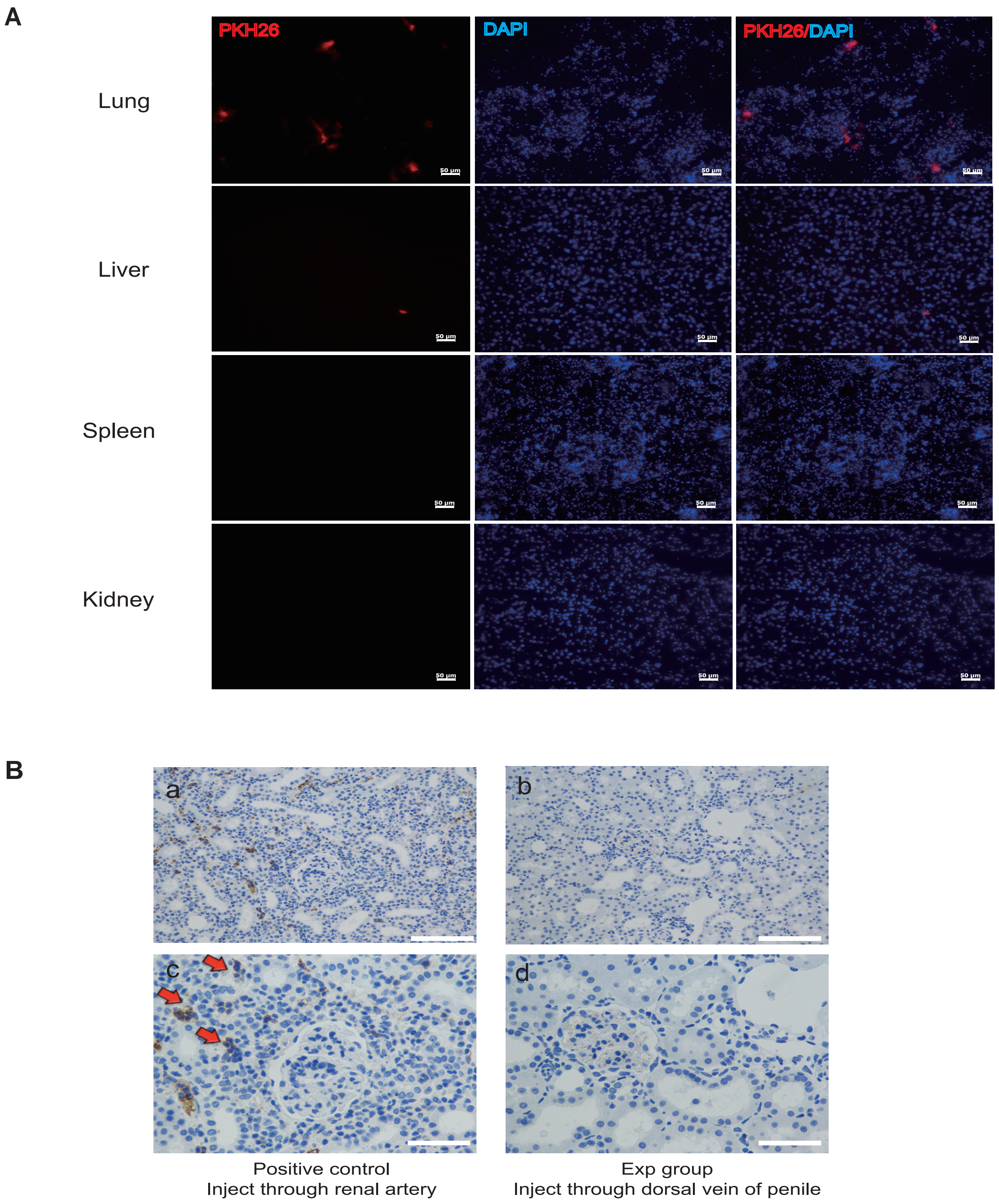
A
Lung
Liver
Spleen
Kidney
PKH26
DAPI
PKH26/DAPI
50 μm
B
a
b
c
d
Positive control
Inject through renal artery
Exp group
Inject through dorsal vein of penile

Figure S1

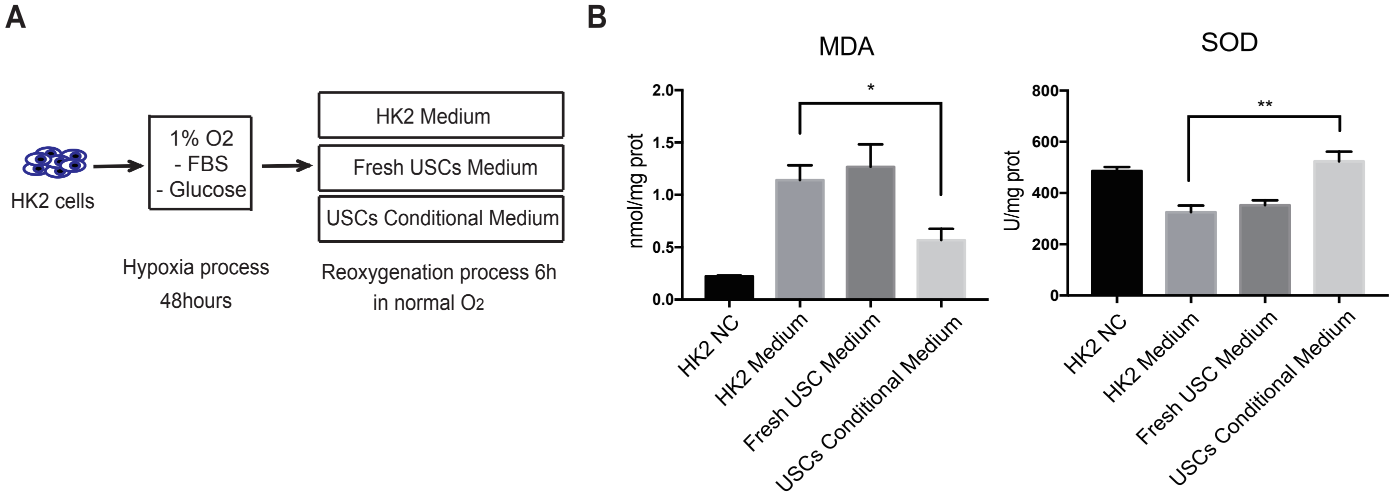
A
HK2 cells
1% O2
- FBS
- Glucose
Hypoxia process
48hours
HK2 Medium
Fresh USCs Medium
USCs Conditional Medium
Reoxygenation process 6h
in normal O2
B
MDA
nmol/mg prot
2.0
1.5
1.0
0.5
0.0
*
HK2 NC
HK2 Medium
Fresh USC Medium
USCs Conditional Medium
SOD
U/mg prot
800
600
400
200
0
**
HK2 NC
HK2 Medium
Fresh USC Medium
USCs Conditional Medium

Figure S2

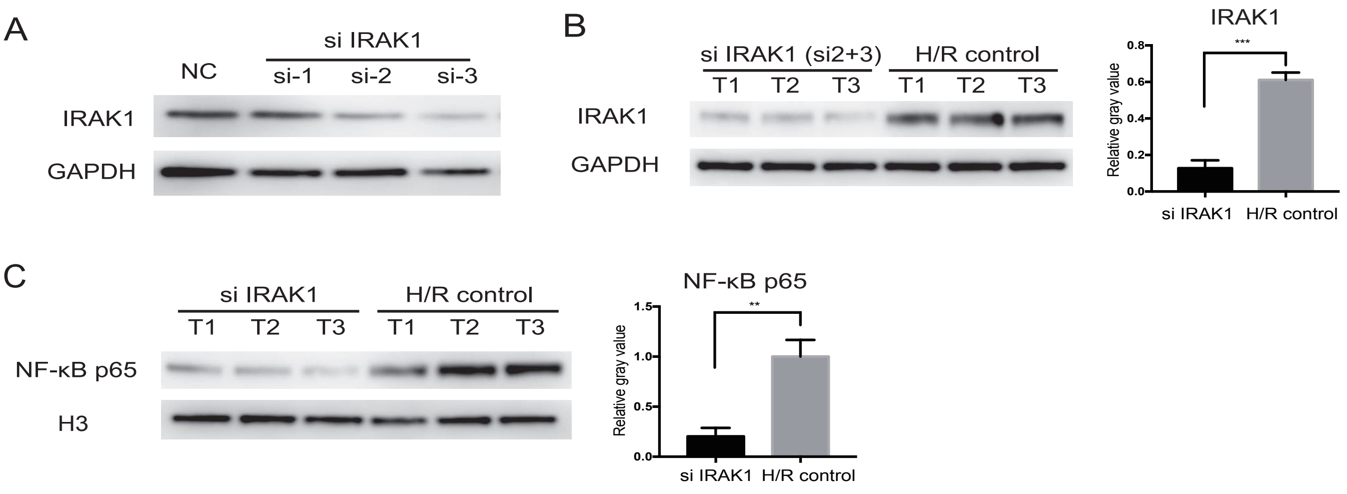
A
si IRAK1
NC
si-1
si-2
si-3
IRAK1
GAPDH
B
si IRAK1 (si2+3)
H/R control
T1
T2
T3
T1
T2
T3
IRAK1
GAPDH
IRAK1
***
0.8
0.6
0.4
0.2
0.0
Relative gray value
si IRAK1
H/R control
C
si IRAK1
H/R control
T1
T2
T3
T1
T2
T3
NF-κB p65
H3
NF-κB p65
**
1.5
1.0
0.5
0.0
Relative gray value
si IRAK1
H/R control

Figure S3
